# Dreams and nightmares during the pandemic

**DOI:** 10.1007/s11818-022-00351-x

**Published:** 2022-05-03

**Authors:** Severin Ableidinger, Franziska Nierwetberg, Brigitte Holzinger

**Affiliations:** 1 Institut für Bewusstseins- und Traumforschung, Canongasse 13/1, 1180 Vienna, Austria; 2 Medizinisches Schlafcoaching, Postgraduate Medizinische Universität Wien, Spitalgasse 23, 1090 Vienna, Austria

**Keywords:** COVID-19, Dreaming and dream recall, Nightmare frequency, Mental health, Trauma, PTSD, COVID-19, Traum und Traumerinnerung, Alptraumfrequenz, Psychische Gesundheit, Trauma, PTBS

## Abstract

The pandemic caused by the coronavirus disease 2019 (COVID-19) had a huge impact on public mental health. This was also reflected in dreams. Not only did people start to remember more dreams, but dream content changed as themes like sickness, confinement, and—in the English-speaking world—even bugs began to dominate. This also led to an increase in nightmare frequency. There are various factors that contributed to this change in the dream landscape. Some people have started to sleep more and hereby spend more time in REM sleep, which is known to increase dream recall and further lead to bizarre and vivid dreams. On the other hand, stress and poor mental health had an impact on sleep, and sleep quality thus dropped in many individuals. Poor sleep quality can also lead to an increase in dream recall. Dreams are known to regulate mood, so the rise in dreams and the change in dream content could also reflect a reaction to the overall rise in stress and decline in mental health. Recent studies have shown that as the pandemic progresses, further changes in mental health, dream recall, and dream content arise, but data are still scarce. Further research could help understand the impact the pandemic still has on mental health and dreams, and how this impact is changing over the course of the pandemic.

## Abstract (de)

Die Pandemie rund um die Coronavirus-Krankheit-2019 (COVID-19) zeigte starke Auswirkungen auf die psychische Gesundheit. Dies wurde auch in den Träumen reflektiert. Nicht nur konnten sich Personen an mehr Träume erinnern, auch deren Inhalte änderten sich. Krankheiten und Einschließungen kamen vermehrt in Träumen vor und im englischsprachigen Raum wurde sogar von Käfern geträumt. Ebenso kam es zu einem Anstieg von Albträumen. Dabei gibt es viele verschiedene Faktoren, über die die Pandemie Träume beeinflusst. Manche Personen konnten mehr schlafen und hatten dadurch mehr REM-Schlaf. Dieser könnte nicht nur für die erhöhte Traumerinnerung verantwortlich sein, sondern auch für das Auftreten von bizarren und lebhaften Träumen. Andererseits verursachten der durch die Pandemie ausgelöste Stress und die damit einhergehende verringerte psychische Gesundheit auch Schlafprobleme, und die Schlafqualität sank bei vielen. Dies kann wiederum auch einen Anstieg der Traumerinnerung verursachen. Da Träume einen Mechanismus zur Emotionsregulation darstellen, könnten aber auch der Stress und die psychische Gesundheit selbst die Auslöser für ein vermehrtes Vorkommen von Träumen sowie deren Veränderung sein. Mit dem Fortschreiten der Pandemie verändern sich auch weiterhin die psychische Gesundheit, die Traumerinnerung und Trauminhalte, allerdings gibt es dazu bisher nur wenig Datenmaterial. Um die noch bestehenden Auswirkungen der Pandemie auf die psychische Gesundheit und auf Träume sowie deren weiteren Verlauf zu verstehen, sind weiterführende Erhebungen notwendig.

## Introduction

In the year 2019, a new respiratory disease was discovered in Wuhan, China. From there, this disease quickly spread across the globe and led to the declaration of a pandemic by the World Health Organization (WHO) in March 2020 [39]. In response to the rising number of cases, various countries proposed different measures to fight and contain the disease. Lockdowns and social distancing were introduced, and as a result, many children had to be homeschooled and a lot of work was done via home office, while some were forced to not work at all or their workload and time was reduced [7]. In the EU, almost half of the population had a reduction in their working hours [7] and over a quarter lost their job or contract permanently or temporarily [7]. This had various consequences. Many people did not have a steady income and had to worry about their financial situation [7]. Especially people between the ages of 35 and 49 and women found it difficult to make ends meet. With their work being done from home and many leisure activities being rendered unavailable, many people found themselves with hardly any social contact left. Every seventh person stated feeling lonely most or all of the time [7]. The disease itself was also a huge stressor, as people feared being sick. Especially for people at a higher risk, like older people or people with preconditions, an infection could be a matter of life or death [40]. Consequently, people not only had to fear for their own physical wellbeing but also for that of their loved ones.

All of this had a huge impact on mental health [5, 7, 22, 41]. Studies have shown that rates of anxiety, depression, insomnia [22], and even PTSD [5] increased. In a meta-analysis, it was shown that after any pandemic, including COVID-19, there are higher rates of PTSD, as high as 22.6% [41]. A different meta-analysis produced similar results and concluded that 23.88% of the population are suffering from PTSD [6]; however, another recent meta-analysis stated that about 15% of the general population suffer from PTSD during the pandemic [42]. This number is still relatively high compared to the lifetime prevalence of PTSD that was calculated to be 3.9% using data gathered by WHO World Mental Health Surveys [15]. In a survey by Eurofound [7], it was found that mental wellbeing is further declining as the pandemic progresses.

## Impact on dreams

The continuity hypothesis of waking and dreaming states that our daily lives get reflected in our dreams and especially significant events and emotional traumas become incorporated [32]. Therefore, it is not surprising that the pandemic had a drastic impact on dreams [8, 27]. This opened a public debate. Internet sites have been created to collect those dreams (for an example, see [12]) and various news outlets picked up on the topic. Prominent examples were CNN [17], the Guardian [18], and the Washington Post [38].

Dream content changed in relation to normative dream content from before the pandemic [2]. People reported dreaming about sicknesses, COVID-19, and confinement, but dreams also incorporated themes like war, totalitarianism, death, and the apocalypse [14]. Dreams involving bugs were also reported. The dream researcher Deirdre Barrett [2] interpreted these dreams as a symbolic expression for the sickness, as being sick is sometimes called “catching a bug” (for an overview about symbolic expression and metaphors in dreams, see [19]).

In an early study conducted in the USA at the beginning of May 2020, about 8.15% of the participants stated to have dreamt about COVID-19 [33]. In a study a little later, conducted in Brazil between May and June 2020, about 33% of the participants reported dreams about COVID-19 [23]. In a more recent American study that took place in the fall of 2020, the proportion of dream reporters, who had a dream about COVID-19 increased to 45–65% [9]. Dream content also seems to change as the pandemic progresses. After a lockdown in Italy ended, people started to dream about crowded places [30].

But not only dream content changed. With the start of the pandemic, both dream recall and nightmare frequency spiked [8, 27]. In a study conducted in May 2020, 26% reported experiencing more nightmares [27]. In another early study, 29% reported having more dreams, while 7.5% reported a decrease in dreams. Further, 15% reported their dreams to get more negative, while 7% reported having more positive dreams [33]. During the early stage of the pandemic in Brazil, a rise in nightmares was reported in 16% of participants [23]. A multinational study found that the proportion of people who reported remembering dreams on three or more nights per week increased by 9% [8]. Also, participants with COVID-19 infections reported even higher rates of nightmares than others without an infection [31]. In one study comparing two timepoints, the first during a lockdown near the beginning of the pandemic in April 2020, and the second during a partial lockdown later in November 2020, at both timepoints, some of the participants stated experiencing changes in dream frequency. In April, 30.5% stated an increase and 21.8% a decrease, while in November, 30.3% stated an increase and 13.5% a decrease in dream frequency [5]. In a study comparing lockdown measures with non-lockdown measures, it was found that during lockdown, dream recall was higher than when there was no lockdown [30]. Also, it should be noted that dream recall and nightmare frequency seem to be associated [8].

## Making sense of changed dreams

But why do people remember more dreams? There are various factors and theories on dream recall, but including all of them would go beyond the scope of this paper. Thus, in this paper, various factors that were found in the cited studies will be mentioned. For further factors and theories relevant for dream recall, look at [34]. One possible explanation for people remembering more dreams is that people could be getting more sleep. As people started working from home, and sometimes had reduced workloads, people didn’t have the need to commute to work, school, or university anymore. Having spared this time, many people could now afford to sleep in. This translated into people reporting longer sleep times [27] and better sleep quality [13]. Interestingly, many did not change their time for going to bed, even though some went to bed later now, but rather changed their time for waking up and getting out of bed, and in this manner increased their total bedtime [1]. This has the effect of enhancing REM sleep, as the proportion of REM sleep in one sleep cycle increases in the second half of the night and before waking up [24]. So the relative amount of REM sleep gets larger later in the night, or better said, later in the total time slept, as not all sleep solely during the night. Even though it is now revoked that REM sleep is equivalent to dreaming and dream research has shown that dreams also occur in non-REM sleep, REM sleep dreams are characterized by being much more vivid, bizarre, and emotional as compared to non-REM sleep dreams [20]. It is therefore plausible that longer bedtime and more sleep is responsible for higher dream recall. But not everybody is getting more sleep. In fact, many studies report insomnia, lower sleep quality, and more awakenings, which should reduce the total sleep time instead of increasing it [22, 25, 27].

In a multinational study, worse sleep quality was found in about 20%, while better sleep quality was found in only 5% [25]. A study comparing sleep quality during and after a lockdown found that 46.07% reported low sleep quality during the lockdown. After the lockdown, people had significantly greater ease falling asleep and fewer awakenings during the night [30]. A different study found similar results, as people experienced longer sleep latency and more difficulties falling asleep during lockdown [1]. However, other research found that sleep quality did not increase after the end of a lockdown [11]. Indeed, it has been found that sleep quality and dream recall are associated, as people with lower sleep quality remembered dreams more frequently [8]. These results received further support as a recent study found dream recall and nightmare frequency to be solely associated with bad sleep quality and not with daily worries about COVID-19 [36].

Many different factors contribute to sleep quality, ranging from stress, physical activity, and physical health, to various psychological factors [3, 16]. Since many people experience a lot of stress during the pandemic, it is logical to expect their sleep quality to decline. Additionally, it has been suggested that people neglect their sleep hygiene, by drinking more alcohol [26] or having longer screen time, even before sleeping [29].

Another factor related to dreaming is mental health. Although the existing literature is not conclusive on the effect of overall mental health and dream recall, it has been shown that stress is related to dream recall [35] and psychopathology to nightmare frequency [28]. As mentioned before, the stressors of the pandemic had a huge impact on public mental health. This translated into a rise in anxiety, depression, insomnia, and PTSD symptoms [5, 22]. Research has shown that heightened dream recall during the pandemic was especially pronounced in people with poorer mental health [8]. But not everyone was affected equally by the pandemic. Mental health problems and high dream recall were especially frequent in women and younger people [8, 22]. COVID-19 infections also increase the risk for anxiety, depression, and PTSD [31]. Additional pandemic-related factors, like financial burden, social isolation, and subjective risks have also been identified to contribute to mental health and dream recall during the pandemic. It has been shown that those who were more severely affected also suffered worse mental implications, like a heightened risk for insomnia [22], and experienced the biggest changes in their dreams and dream content [33]. Further, anxiety and PTSD are known to have a connection to nightmares [21]: 80% of PTSD patients suffer from regular nightmares [21]. So the rise in symptoms of both anxiety and PTSD could also contribute to the rise in dream recall and nightmares. Nightmares themselves can also lead to various disturbances. Decline in sleep quality, mood disturbances, daytime sleepiness, and cognitive impairment are some of the harmful results nightmares can bring [21]. In the context of the pandemic, this could indicate a vicious circle, where daily stress leads to nightmares, which themselves further lead to various problems and more daily stress.

Dreams are often seen as a mechanism that regulates our mood [4, 37]. Therefore, increased dream recall could further indicate a natural way to deal with the overall stress during the pandemic. This would also be consistent with studies reporting changes in dream frequencies after other significant and threatening events (see [24]).

## Conclusion

The pandemic has had a huge impact. It has affected mental health and caused various psychological symptoms. Further, it has influenced dream content and led to a rise in dream recall. As dreams started to get more negative, themes of sicknesses and pandemics arose, and nightmare frequency thus also spiked. This demonstrates how our daily lives influence our dreams, and how they reflect our mental health and wellbeing. Further, the quantity of changes shows how much of an impact the pandemic has had on the general population. This also implies that sudden changes in dream recall and dream content can reflect significant events and changes in mental health and sleep quality. So assessing dreams and dream frequency could help detect changes in mental wellbeing in the population.

Although the cited studies seem to show that the overall dream landscape has changed, it is necessary to emphasize that for all of the studies, participation was voluntary. Therefore, especially people who already had a change in dream and nightmare frequency as well as dream content could have chosen to participate in these studies which focused on dreams and COVID-19. However, the study by Callagher and Incelli [9] tested especially whether this sort of self-selecting bias leads to an overestimation of the changes in dream and nightmare frequency and dream content, or even creates the described changes in the first place. They did not give their participants any information about the purpose of the study until participation and yet they still found the described changes. However, all of the studies comparing pre-pandemic to pandemic dreams ask retrospectively, so there could possibly be memory bias.

In this article the continuity theory of dreaming has already been mentioned, as has emotion regulation, which is also central to various theories of dreaming. However, there are many more theories regarding the purpose of dreams. A complete listing of all theories and models would go beyond the scope of this review, for further reading [10, 19] are referred to.

As of now, most studies have explored the impact at the beginning of the pandemic, but more recent research has shown that there are differences between lockdown and post-lockdown periods, and that both are different to pre-pandemic times. But data on how dreams change during progression and time course of the pandemic are still lacking. It seems as if mental health problems get worse the longer the pandemic progresses, and this should also be reflected in dreams. So assessing dreams could help in finding trends regarding how mental health and wellbeing change throughout the course of the pandemic. Further, as dreams work as an emotional regulation mechanism, assessing nightmare and dream frequency could help assess whether this mechanism still functions properly and indicate heightened emotional load.

## Declarations

For this article no studies with human participants or animals were performed by any of the authors. All studies mentioned were in accordance with the ethical standards indicated in each case.
